# Advances in the research of sulfur dioxide and pulmonary hypertension

**DOI:** 10.3389/fphar.2023.1282403

**Published:** 2023-10-12

**Authors:** Xin Liu, He Zhou, Hongsheng Zhang, Hongfang Jin, Yan He

**Affiliations:** ^1^ Department of Pediatric Cardiac Center, Beijing Anzhen Hospital, Capital Medical University, Beijing, China; ^2^ Departments of Medicine and Physiology, Tulane University School of Medicine, New Orleans, LA, United States; ^3^ Department of Pediatrics, Peking University First Hospital, Beijing, China; ^4^ State Key Laboratory of Vascular Homeostasis and Remodeling, Peking University, Beijing, China

**Keywords:** pulmonary hypertension, sulfur dioxide, aspartate transaminase, pulmonary vascular remodeling, gaseous signaling molecule

## Abstract

Pulmonary hypertension (PH) is a fatal disease caused by progressive pulmonary vascular remodeling (PVR). Currently, the mechanisms underlying the occurrence and progression of PVR remain unclear, and effective therapeutic approaches to reverse PVR and PH are lacking. Since the beginning of the 21st century, the endogenous sulfur dioxide (SO_2_)/aspartate transaminase system has emerged as a novel research focus in the fields of PH and PVR. As a gaseous signaling molecule, SO_2_ metabolism is tightly regulated in the pulmonary vasculature and is associated with the development of PH as it is involved in the regulation of pathological and physiological activities, such as pulmonary vascular cellular inflammation, proliferation and collagen metabolism, to exert a protective effect against PH. In this review, we present an overview of the studies conducted to date that have provided a theoretical basis for the development of SO_2_-related drug to inhibit or reverse PVR and effectively treat PH-related diseases.

## 1 Introduction

Pulmonary hypertension (PH) is an abnormal hemodynamic state with a mean pulmonary arterial pressure (mPAP) > 20 mmHg at rest, as measured by right heart catheterization (Simonneau et al., 2019). The progressive structural changes in pulmonary vasculature are the underlying cause of its life-threatening nature. With the increasing global aging population and extended survival of individuals with congenital heart and lung mal-formations, approximately 1% of the world’s population is affected by PH (Mandras et al., 2020). Therefore, elucidating the mechanisms of PH development and identifying effective strategies to prevent or reverse pulmonary vascular remodeling (PVR) are crucial topics in cardio-pulmonary vascular research. Previous studies have revealed that pulmonary vascular structural alterations are intricately regulated by various factors, including pulmonary arterial forward flow, vasoactive substances (endothelin [ET]/endostatin, nitric oxide [NO], hormones, vascular endothelial growth factor, platelet-derived growth factor and transforming growth factor [TGF]) and genetic factors (Haworth and Reid, 1977; Benza et al., 2015; Liang et al., 2022; Zhou et al., 2022; Zhu et al., 2022; Eichstaedt et al., 2023). These factors can exert differential effects at different levels and degrees on the pathological processes of endothelial cell (EC) and smooth muscle cell (SMC) proliferation, migration and differentiation within the pulmonary vasculature. However, the precise mechanisms underlying the development of PH remain poorly understood.

In 2008, Du and Jin et al. (Du et al., 2008) reported the existence of an endogenous sulfur dioxide (SO_2_) and aspartate transaminase (AAT) pathway in rat blood vessels. This discovery expanded the research of the gasotransmitter family (NO, carbon monoxide, hydrogen sulfide [H_2_S] and SO_2_) in the field of PH, in which the SO_2_ pathway became a novel finding (Jin et al., 2008; Tian et al., 2008). SO_2_ has the biological characteristics of gasotransmitters, including sustained production, rapid diffusion, a short half-life, an ability to pass through cell membranes freely and more. Furthermore, it exerts various biological effects on the cardiovascular system and regulates pathological and physiological activities, such as cellular inflammation, apoptosis, proliferation, and collagen metabolism (Yu et al., 2016; Shi et al., 2020; Tian et al., 2020; Zhu et al., 2020). In recent years, the in-depth research on endogenous SO_2_ in the vascular system has provided novel insights into the mechanisms underlying the development of PH. This review summarizes the current progress in the study of SO_2_ and PH.

## 2 Metabolism and distribution of SO_2_ in pulmonary vasculature

The pulmonary vascular wall consists of three layers: the intima, media, and adventitia which are primarily composed of ECs, SMCs and fibroblasts respectively (Huertas et al., 2020). Additionally, perivascular adipose tissue (PVAT), a specialized type of fat tissue, surrounds the adventitia of pulmonary blood vessels and is primarily composed of adipocytes. Furthermore, circulating cells like mast cells (MCs) and monocytes/macrophages can cross the pulmonary vascular wall, and previous studies have shown that these cells can produce various vasoactive substances that maintain the pulmonary vascular homeostasis (Breitling et al., 2017; Tinajero and Gotlieb, 2020; Hudson and Farkas, 2021; Li et al., 2021; Chen et al., 2022). Recent studies have revealed that endogenous SO_2_ production occurs continuously in these cell populations (Liu et al., 2016; Yu et al., 2016; Zhang et al., 2016; Zhu et al., 2020; Liu et al., 2021; Zhang et al., 2021).

### 2.1 Synthetic metabolic pathways of SO_2_

**FIGURE 1:**
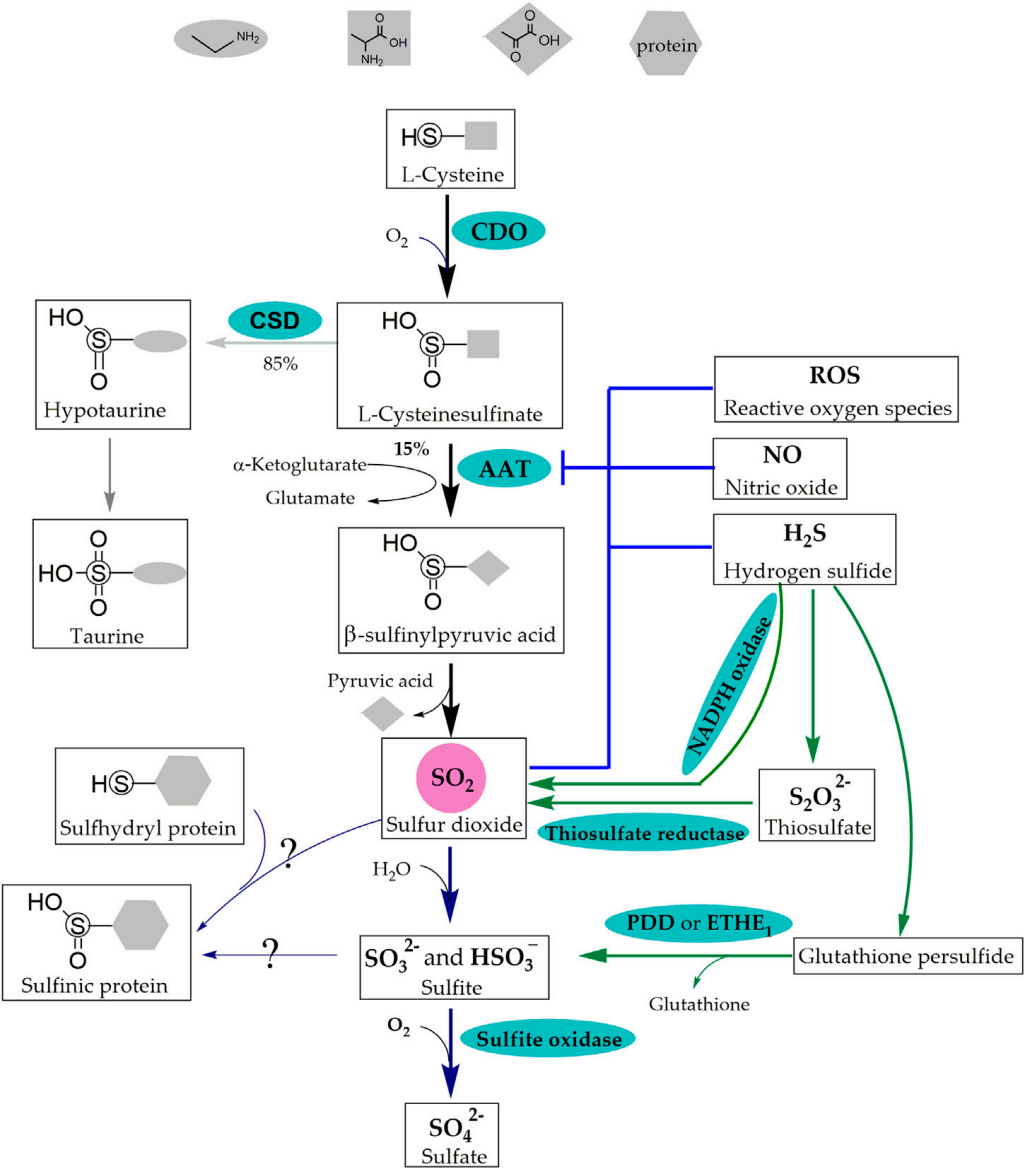
Synthetic Metabolic and Catabolic Pathways of SO_2_. The sulfur-containing amino acid metabolic pathway and the conversion of H_2_S are the two pathways of endogenous SO_2_ production. CDO catalyzes L-cysteine to generate L-cysteinesulfinate, 15% of which is transformed into β-sulfinylpyruvate through AAT that is inhibited by ROS, NO, H_2_S and SO_2_. β-Sulfinylpyruvate then decomposes naturally into pyruvate and SO_2_ in the sulfur-containing amino acid metabolic pathway. In addition, NADPH oxidase, thiosulfate reductase and PDO or ETHE1 are involved in the conversion of H_2_S into SO_2_. Molecular SO_2_ is rapidly converted to HSO_3_^−^ and SO_3_^2-^, which further transform into SO_4_^2-^ by sulfite oxidase or form -S (=O)-OH in protein containing thiol groups. SO_2_: sulfur dioxide; H_2_S: hydrogen sulfide; CDO: cysteine dioxygenase; AAT: aspartate aminotransferase; ROS: reactive oxygen species; NO: nitric oxide; NADPH: nicotinamide-adenine dinucleotide phosphate; PDO or ETHE1: persulfide dioxygenase; HSO_3_^−^ and SO_3_^2−^: sulfite; SO_4_^2−^: sulfate; -S (=O)-OH: sulfinic acid groups. CSD: cysteinesulfinate decarboxylase; S_2_O_3_^2−^: thiosulfate.

The endogenous production of SO_2_ can occur through two pathways: the sulfur-containing amino acid metabolic pathway and the conversion of H_2_S; the sulfur-containing amino acid (primarily including methionine, cysteine, and homocysteine [Hcy]) pathway has been extensively studied and is considered as the primary source of SO_2_ (Figure 1). This pathway has been identified in various cell types within the pulmonary vascular wall, such as ECs, SMCs and fibroblasts (Liu et al., 2016; Yu et al., 2016; Liu et al., 2021).

#### 2.1.1 Generation of endogenous SO_2_ via the sulfur-containing amino acid metabolic pathway

In the cytoplasm, L-cysteine derived from dietary intake or synthesized via transsulfuration is converted into L-cysteinesulfinate by the catalytic action of cysteine dioxygenase (CDO) (Yamaguchi et al., 1973; Kohashi et al., 1978). Subsequently, 15% of the L-cysteinesulfinate is further transformed into β-sulfinylpyruvate through AAT, which possesses cysteine sulfinic acid transaminase (CSA-T) activity. Finally, β-sulfinylpyruvate decomposes naturally into pyruvate and SO_2_ (Singer and Kearney, 1956; Yagi et al., 1979; Griffith, 1983; Stipanuk, 2020).

AAT is the key rate-limiting enzyme in the endogenous generation of SO_2_ and has two isoenzymes, AAT1 and AAT2. Immunohistochemical localization in rat brain tissue revealed that AAT1 and AAT2 were localized in the cytoplasm and mitochondria, respectively (Recasens et al., 1980; Recasens and Delaunoy, 1981).

Studies on the regulation of AAT activity and expression have revealed a competitive relationship between cysteinesulfinate decarboxylase (CSD) and AAT. Approximately 85% of L-cysteinesulfinate undergoes decarboxylation by CSD to produce taurine. Treatment with β--methylene-DL-aspartate, an irreversible inhibitor of CSD, resulted in an approximately three-fold increase in AAT transamination; however, changes in the levels of SO_2_ were not detected (Griffith, 1983).

Negative feedback inhibition is the most common regulatory mechanism in organisms. Song et al. (2020) reported that SO_2_ did not affect the protein expression of AAT1 but significantly inhibited AAT activity in purified porcine AAT1 protein. Further investigation revealed that SO_2_ inhibited AAT activity in ECs by sub-sulfinylation at the Cys192 site of AAT1.

There is a close interconnection between gasotransmitter molecules. In regard, Zhang et al. (2018) performed *in vitro* experiments using the human umbilical vein endothelial cell (HUVEC) line EAhy926, primary HUVECs, primary rat pulmonary artery endothelial cells (PAECs), and purified AAT protein from porcine heart. They discovered that the knockdown of cystathionine γ-lyase (CSE) resulted in a decrease in endogenous H_2_S level accompanied by an increase in EC-derived SO_2_ level and AAT enzyme activity. However, it did not affect the expression of AAT1 and AAT2 and the alterations in the SO_2_/AAT pathway could be reversed by H_2_S donor. Mechanistic studies revealed that H_2_S inactivated AAT1/2 via cysteine modification to inhibit endogenous SO_2_ generation. In 2021, Song et al. (2020) reported that in L-NAME-induced hypertensive mice, plasma NO level was reduced, whereas SO_2_ level was elevated in L-NAME-induced hypertensive mice. Furthermore, in a coculture system of human aortic endothelial cells (HAECs) and human aortic smooth muscle cells (HASMCs), the HAEC-derived NO did not affect AAT1 protein expression but decreased AAT1 activity in HASMCs, consequently inhibiting HASMC-derived SO_2_ production. Liquid chromatography with tandem mass spectrometry demonstrated that NO inhibited AAT1 activity by S-nitrosylation at the Cys192 site of purified AAT1 protein. Reactive oxygen species (ROS) modulate the activities of various enzymes through redox modification. *In vitro* experiments with vascular SMCs showed that the ROS scavenger N-acetyl-L-cysteine and antioxidant glutathione (GSH) significantly abolished the downregulation of the SO_2_/AAT pathway induced by ET-1 stimulation (Tian et al., 2020), suggesting that ROS inhibited the endogenous SO_2_/AAT pathway.

#### 2.1.2 Endogenous generation of SO_2_ via H_2_S transformation

H_2_S transformation into SO_2_ occurs in three ways: 1) nicotinamide-adenine dinucleotide phosphate oxidase (NADPH oxidase) participates in an oxidative stress reaction, which converts H_2_S into SO_2_. This process was observed in activated neutrophils (Mitsuhashi et al., 2005); 2) H_2_S generates thiosulfate in a non-enzymatic reaction and cytoplasmic thiosulfate reductase catalyzes the conversion of thiosulfate (S_2_O_3_^2-^) to SO_2_ (Kamoun, 2004; Cao et al., 2019); 3) mitochondrial persulfide dioxygenase (PDO or ETHE1) catalyzes the conversion of non-enzymatically generated glutathione persulfide (GSSH) derived from H_2_S into sulfite (SO_3_^2-^) and glutathione (GSH) (Hildebrandt and Grieshaber, 2008; Kabil et al., 2018; Stipanuk, 2020).

### 2.2 Catabolic pathways of SO_2_

In 1995, Ji et al. (1995) reported that the concentration of SO_3_^2−^ in normal human serum, as detected by high-performance liquid chromatography (HPLC), was significantly low, ranging from 0 to 9.85 μM. Subsequently, the research group headed by Du and Jin were the first to report the presence of SO_3_^2−^ (3.27 ± 0.21 µmol/[gprotein]) in rat pulmonary vascular tissue (Du et al., 2008). The findings of previous studies have thus indicted that in body fluids, a larger proportion of molecular SO_2_ is rapidly converted to its hydrated form (HSO_3_^−^ and SO_3_^2−^), which is further converted to sulfate (SO_4_^2−^) under the catalytic action of sulfite oxidase (Figure 1) (Singer and Kearney, 1956). SO_4_^2-^ is then excreted through the kidneys to maintain the metabolic homeostasis of sulfur-containing amino acids in the body (Stipanuk, 2020). However, recent research has reported that the lifetime of gasotransmitters may be much longer than those previously estimated (Elrod et al., 2008; Bahadoran et al., 2020). Furthermore, body fluid contains significant amounts of protein, such as plasma albumin and functional proteins containing thiol groups. The application of exogenous SO_2_ donor indicated that SO_3_^2−^ could react with functional proteins containing thiol groups to form proteins containing sulfinic acid groups (-S [ = O]-OH); however, the enzymes involved in this reaction have not been investigated (Chen et al., 2017). These findings suggest that molecular SO_2_, SO_3_^2−^ and proteins containing “-S (=O)-OH” in body fluid may function as a whole.

### 2.3 Distribution of SO_2_ in the pulmonary circulation

Considering that SO_3_^2−^ may be the primary form of endogenous SO_2_ in the pulmonary circulation, current researchers primarily focus on detecting SO_3_^2−^ using HPLC for the analysis of lung tissues or fluorescence probe-based detection in cultured pulmonary vascular cells to indicate the level of SO_2_ (Du et al., 2008; Sun et al., 2010; Liu et al., 2021; Zhang et al., 2021). However, given that SO_2_ fluorescent probe cannot be used to measure SO_3_^2-^ in tissues (Huang et al., 2022), the distribution of SO_2_ in the vessels of animal tissues was indirectly assessed via *in situ* hybridization, immunohistochemistry, and immunofluorescence to detect the mRNA and protein expression of the key enzyme AAT, which indirectly reflected the differential distribution of SO_2_ in the vasculature (Du et al., 2008; Liu et al., 2021).

#### 2.3.1 Tissue distribution

In adult rat cardiac tissue, the mRNA, protein expression and activity of AAT1 and AAT2 were significantly higher than those in the lung tissue. In contrast, however, the concentration and production rate of SO_2_ in the heart were not significantly higher than those in the lung, suggesting that tissue SO_2_ content did not exhibit a simple linear relationship with AAT expression and activity (Luo et al., 2011). This in turn provides evidence to indicate that tissue SO_2_ content may be subject to more stringent regulation or alternatively, is influenced by circulating SO_2_. Du et al. (2008) investigated the endogenous production of SO_2_ and the expression of AAT mRNA in different rat vascular tissues, providing initial evidence of the existence of endogenous SO_2_ within the vascular system. Indirect analysis of endogenous SO_2_ level in different rat vascular tissues was performed by measuring HSO_3_^−^ and SO_3_^2−^ content using HPLC. The results showed that the aortic tissue exhibited the highest level of endogenous SO_2_ among the rat vascular tissues, followed by the pulmonary, mesenteric, tail and renal arteries. Notably, the relationship between the AAT activity and SO_2_ content was non-linear. Using a colorimetric method to determine AAT activity, Du and Jin et al. (Du et al., 2008) reported that AAT activity was the highest in the renal artery, followed by the tail, mesenteric, pulmonary and aorta arteries. However, given the limitations of the SO_2_ detection methods and the diversity of SO_2_ chemical forms, these results indicating changes in AAT activity in different vascular tissues may not necessarily reflect the actual changes in SO_2_ content. In recent research, SO_2_ content in adipose tissue was measured using HPLC and the localization and qualitative expressions of the mRNA and protein of AAT1 and AAT2 were investigated. These results demonstrated the presence of the SO_2_/AAT pathway in various adipose tissues of rats, including the PVAT and perirenal, epididymal, subcutaneous, and brown adipose tissues (Zhang et al., 2016).

#### 2.3.2 Cellular distribution

Previous studies focusing on the distribution of SO_2_ in vascular tissues revealed a predominant distribution of AAT1 and AAT2 mRNA in ECs and SMCs near the intima in the rat aorta by *in situ* hybridization (Du et al., 2008). Subsequently, immunofluorescence analysis of mouse lung tissue sections showed that the highest expression of AAT1 protein was in PAECs. Furthermore, the detection of HSO_3_^−^ and SO_3_^2-^ using SO_2_ probe indicated the endogenous production of SO_2_ in human PAECs (HPAECs) (Liu et al., 2021). The presence of the endogenous SO_2_/AAT pathway in pulmonary vascular SMCs and fibroblasts involved in PVR was demonstrated by HPLC, SO_2_ probes, and Western blot experiments (Liu et al., 2016; Yu et al., 2016).

Additionally, the endogenous SO_2_/AAT pathway is present in cell types that those are closely associated with PVR, such as perivascular adipocytes, MCs and macrophages. Analysis using HPLC and Western blot demonstrated the presence of endogenous SO_2_/AAT1 pathway in 3T3-L1 adipocytes (Zhang et al., 2016). Zhu et al. (2020) detected the endogenous production of SO_2_ in mouse macrophages using HPLC and SO_2_ probe; moreover, Western blot analysis confirmed the mouse macrophage-derived protein expression of AAT2. Latterly Zhang et al. (2021) reported the existence of endogenous SO_2_/AAT1 pathway in human MCs *in vitro*.

## 3 Metabolic changes of SO_2_ in PH

**FIGURE 2:**
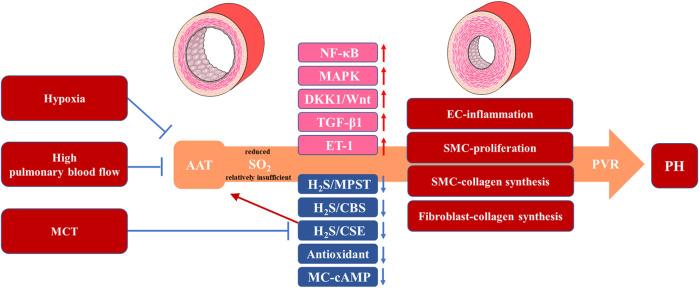
The Metabolic Changes and Protective Roles of SO_2_ in Animal PH Models. Animal experimental results suggest a reduced or relatively insufficient endogenous SO_2_ production mediated by AAT under hypoxia, high pulmonary blood flow and MCT, which promotes or suppresses signaling pathways (NF-κB, MAPK, DKK1/Wnt, TGF-β1, H_2_S/MPST/CBS/CSE and MC-cAMP) and vasoactive substances (ET-1 and antioxidant) associated with the pathological progress in EC-inflammation, SMC-proliferation, SMC-collagen synthesis and fibroblast-collagen synthesis. These pathological processes cause PVR manifesting as a thickening of the pulmonary vascular wall and PH with an mPAP >20 mmHg. SO_2_: sulfur dioxide; PH: pulmonary hypertension; AAT: aspartate aminotransferase; MCT: monocrotaline; H_2_S: hydrogen sulfide; 3-MPST: 3-mercaptopyruvate sulfurtransferase (3-MPST); CBS: cystathionine β-synthase; CSE: cystathionine γ-lyase; MC: mast cell; ET-1: endothelin-1; EC: endothelial cell; SMC: smooth muscle cell; PVR: pulmonary vascular remodeling; mPAP: mean pulmonary arterial pressure.

Pulmonary arterial pressure gradually decreases after birth. Factors, such as respiration, lung expansion and the closure of ductus arteriosus, contribute to a decline by approximately 50% in pulmonary vascular resistance and pulmonary arterial pressure. This results in increased pulmonary blood flow, which contributes to the dilation of pulmonary blood vessels and a progressive increase in the number of pulmonary arterioles. Consequently, the luminal diameter of the pulmonary arterioles expands and the vessel walls become thin leading to a sustained decrease in pulmonary arterial pressure (Hislop, 2002). However, ventricular septal defects, pulmonary developmental abnormalities, abnormal lung development or inflammation in patients with chronic obstructive pulmonary disease (COPD) can impede or reverse the decline in pulmonary arterial pressure through constriction of pulmonary blood vessels, reduction in the number of pulmonary vessels and PVR (Bonafiglia et al., 2022; Golovenko et al., 2022; Kovacs et al., 2022). As the gasotransmitter family has gained increasing attention and recognition in PVR research, the first member, NO has been successfully applied in the clinical treatment of PH (Ziegler et al., 1998; Creagh-Brown et al., 2009) and the novel gasotransmitter SO_2_ has recently garnered increasing focus on its metabolic changes in PH (Figure 2) (Jin et al., 2008; Luo et al., 2013; Huang et al., 2016; Huang et al., 2022).

### 3.1 Metabolism of SO_2_ in clinical PH

Limited research has been conducted on endogenous SO_2_ in clinical PH and has been restricted to children with congenital heart disease-associated PH (CHD-PH). In 2014, Yang et al. (2014) conducted a prospective cohort study to investigate the relationship between plasma SO_2_ level and pulmonary arterial pressure in children with CHD-PH. On the basis of HPLC analysis, they discovered that in children with left-to-right shunt CHD, the measured levels of SO3^2-^ in the control, CHD without PH, CHD with mild PH and CHD with moderate-to-severe PH groups were 10.6 ± 2.4, 8.9 ± 2.3, 7.3 ± 2.9 and 4.3 ± 2.1 μM, respectively. These values were consistent with the serum concentration of HSO_3_^−^ and SO_3_^2-^ reported by Ji et al. (1995), which ranged from 0 to 9.85 μM in healthy individuals. Pearson’s correlation analysis showed a negative correlation between SO2 level and pulmonary arterial pressure in all CHD groups. Across all four groups, there was a negative correlation between SO_2_ and Hcy levels. These results suggested a decrease in endogenous SO_2_ level in children with CHD-PH and the degree of SO_2_ downregulation may reflect the severity of CHD-PH to some extent. An elevated Hcy level indicated a close association between reduced SO_2_ and the Hcy-SO_2_/AAT pathway, a metabolic pathway for sulfur-containing amino acids. A nationwide study has reported a linear correlation between serum AAT and age in Middle Eastern children and adolescents (Kelishadi et al., 2012). However, Li et al. (2018) revealed that the reference range for serum AAT based on adult standards did not apply to adolescents and established the reference ranges for serum AAT in Chinese adolescent boys and girls as 12.8–40.2 U/L and 10.4–32.5 U/L, respectively, to guide clinical practice. However, research on the association between human AAT and PH has not yet been published.

### 3.2 SO_2_ metabolism in experimental PH models

Because of the difficulty in obtaining lung tissue specimens from patients with PH in clinical settings, researchers have predominantly utilized various animal models to investigate changes in the SO_2_/AAT pathway during the development of PH. Consistent with the clinical observation of PH, significant changes in the endogenous SO_2_/AAT pathway have been observed in monocrotaline (MCT), hypoxia and high pulmonary blood flow induced PH models.

Jin et al. (2008) were the first to investigate the metabolic changes in the endogenous SO_2_/AAT pathway in the MCT-induced rat PH model. The results showed that compared to the control group, MCT-treated rats exhibited significantly increased mPAP and right ventricle/(left ventricle + septum) ratio, accompanied by elevated SO_2_ content, AAT activity, and AAT1 and AAT2 mRNA expression in the lung tissue. Subsequently, Yu et al. (2016) reported a significant increase in AAT1 protein expression but no significant change in AAT2 protein expression in the lung tissue of MCT-induced rat PH models, suggesting that MCT primarily induced the upregulation of the endogenous SO_2_/AAT1 pathway in the lung tissue. How did MCT induce the upregulation of the SO_2_/AAT1 pathway. Combining previous studies confirming the inhibition of H_2_S generation in rat lung tissues and HPAECs by MCT (Feng et al., 2017), Jin and Du et al. (Zhang et al., 2018) found that a deficiency in endogenous H_2_S caused by the knockdown of CSE in rat primary PAECs increased endogenous SO_2_ level and enhanced the enzymatic activity of AAT without affecting the expression of AAT1 and AAT2. Furthermore, H_2_S donor reversed the increase in endogenous SO_2_ content and AAT activity induced by CSE knockdown. In addition, H_2_S donor directly inhibited the activity of purified AAT1 protein, which was reversed by the thiol-reducing agent dithiothreitol. Collectively, the studies above suggested that MCT initially reduced H_2_S level in rat PAECs, consequently weakening the inhibitory effect of H_2_S on the cysteine modification of AAT1 protein, thereby leading to increased AAT activity and SO_2_ content in PAECs. However, the mechanism underlying the increase in AAT1 protein expression remains unclear; this may be associated with the different experimental periods between animal studies and cell experiments.

In studies on hypoxia-induced PH, (Tian et al., 2008; Sun et al., 2010) detected significant decreases in SO_2_ content, AAT1 and AAT2 mRNA levels, and AAT activity in the plasma and lung tissue of rats with hypoxia-induced PH compared with those in the control group. In mouse hypoxia-induced PH and hypoxic HPAEC models, the downregulation of the SO_2_/AAT1 pathway in mouse lung tissues and PAECs was observed using HPLC and SO_2_ probe to detect SO_2_ concentration, and immunofluorescence and Western blot to evaluate PAEC-derived AAT1 expression (Liu et al., 2021). However, the specific mechanisms underlying hypoxia-induced downregulation of SO_2_/AAT1 pathway remain unclear.

The high pulmonary blood flow model is used to simulate the development of CHD- PH, and in this regard, Luo et al. (2013) found that compared with the sham group, rats in the shunt group exhibited a significant increase in pulmonary artery systolic pressure, accompanied by a significant decrease in SO_2_ concentration, AAT activity and AAT2 protein and mRNA expression levels in the lung tissue. Subsequently, Liu et al. (2016) reported that there were significant reductions in SO_2_ concentration, AAT activity and AAT1 protein expression in a mechanical stretch model of pulmonary artery fibroblasts (PAFs), indicating that excessive pulmonary artery dilation caused by high pulmonary blood flow contributed to the downregulation of the SO_2_/AAT pathway in high pulmonary blood flow-induced rat PH models.

## 4 Protective roles of SO_2_ in PH

PH is a prevalent and life-threatening disease characterized by PVR, involving PAEC dysfunction, pulmonary artery smooth muscle cell (PASMC) and PAF proliferation and collagen accumulation in the pulmonary arteries (Christou and Khalil, 2022; Milara et al., 2022; Veith et al., 2022; Zhang et al., 2022). Previous studies have reported that an imbalance in various vasoactive small molecules, including gasotransmitters, plays a crucial role in PVR progression (Klinger et al., 2013; Sun et al., 2022; Turhan et al., 2022). Additionally, as a newly discovered gasotransmitter, SO_2_ is involved in the regulation of pathophysiological processes in the pulmonary vasculature and aorta (Supplementary Table S1), such as vascular cell inflammation, apoptosis, proliferation, and collagen metabolism (Yu et al., 2016; Shi et al., 2020; Tian et al., 2020; Zhu et al., 2020). There is a growing body of literature comprising studies that have examined the protective role of SO_2_ in animal PH models using exogenous SO_2_ donor or genetically modified mice (Figure 2).

### 4.1 Hypoxia-induced PH models

Varying degrees of hypoxia occurs in the lungs of patients with PH caused by COPD, connective tissue disease or other conditions. Hypoxia is the most significant pathogenic factor contributing to PVR and the development of PH (Stenmark et al., 2006). Therefore, researchers frequently employ the principle of hypoxia to develop animal PH models.

Tian et al. (2008) investigated the effect of SO_2_ on PVR in a rat model of hypoxia-induced PH. They discovered that exogenous SO_2_ donor could suppress the elevation of mPAP induced by hypoxia and improve changes in the relative medial thickness of muscular arteries and the ultrastructure of PAECs and SMCs. Subsequently, the cellular and molecular mechanisms involved in the inhibition of PVR by endogenous and exogenous SO_2_ in the rat model of hypoxia-induced PH were explored by Chen and Sun independently. By focusing on the interaction among sulfur-containing gasotransmitters, Chen et al. (2011) discovered that providing exogenous SO_2_ donor upregulated the expression of the H_2_S-generating enzyme CSE in the pulmonary artery intima and 3-mercaptopyruvate sulfurtransferase (3-MPST) protein in the pulmonary artery media, leading to increased H_2_S production and indirectly alleviating hypoxia-induced PH. Sun et al. (2010) first employed L-aspartate-β-hydroxamate (HDX) to inhibit endogenous SO_2_ production and demonstrated that HDX exacerbated hypoxia-induced elevation of pulmonary arterial pressure and PVR. The molecular mechanisms involved included inflammation-related factor ICAM-1 and the NF-κB signaling pathway in PAECs, proliferating cell nuclear antigen and the MAPK (Raf-1/MEK-1/ERK) signaling pathway in PASMCs, and ET-1 and collagen metabolism in the lung tissue. Furthermore, the administration of exogenous SO_2_ donor mitigated the aforementioned hypoxia-induced molecular changes, which were consistent with the findings of Tian et al. (Tian et al., 2008; Sun et al., 2010).

After our previous study revealed an association between SO_2_, ICAM-1 and the NF-κB signaling pathway in hypoxia-induced PAEC inflammation (Sun et al., 2010), we further employed transgenic technology to enhance endogenous SO_2_ generation in mouse PAECs. The results showed that, similar to the effects of exogenous SO_2_, endogenous SO_2_ generated by PAECs significantly suppressed the expression of ICAM-1, an inflammation-related factor, in mice with hypoxia-induced PH. At the cellular level, inhibition of the NF-κB signaling pathway attenuated the expression of ICAM-1 and MCP-1 induced by SO_2_ reduction in HPAECs (Liu et al., 2021). Therefore, our findings demonstrated that the NF-κB signaling pathway mediated the inhibitory effect of SO_2_ on the abnormal inflammatory response of PAECs in the development of hypoxia-induced PVR and PH.

Studies on the effects of SO_2_ on PASMC proliferation and collagen metabolism in the context of hypoxia have implicated not only the MAPK signaling pathway involved, but also the Wnt and NF-κB signaling pathways. Members of the Wnt signaling pathway, including Wnt7b, Sfrp2, and Dkk1, participate in vascular cell survival, proliferation, migration and morphogenesis, and Dkk1 inhibits the Wnt signaling (Clevers and Nusse, 2012). In 2018, Luo et al. (Luo et al., 2018) investigated the mechanisms by which SO_2_ inhibits hypoxia-induced PVR. Through RNA-seq analysis of mRNA expression profiles in mouse normal and hypoxic pulmonary arteries, 509 differentially expressed mRNAs were identified. The results obtained in both RNA-seq and PCR analyses provided evidence to indicate the significant downregulation of Wnt7b, Sfrp2 and Cacna1f mRNA expression and a significant upregulation of Dkk1 mRNA expression in the pulmonary arteries of the hypoxic PH group. Interestingly, SO_2_ upregulated Wnt7b, Sfrp2, and Cacna1f mRNA levels and downregulated Dkk1 mRNA level in the pulmonary arteries under hypoxia. Further research confirmed that SO_2_ inhibited hypoxia-induced PASMC migration. To date, the NF-κB signaling pathway has been extensively studied and is involved in the regulation of various cellular processes, including proliferation, inflammation, differentiation and apoptosis (Oeckinghaus et al., 2011; Zhang et al., 2017). Using a mouse model of hypoxic PH, we observed that EC-derived SO_2_ significantly suppressed Ki-67 and collagen I expression in PASMCs. Furthermore, blockade of the NF-κB signaling pathway abolished the effect of SO_2_ reduction on the overexpression of Ki-67 and collagen I in human PASMCs (Liu et al., 2021). These findings suggested that the inhibitory effects of SO_2_ on PASMC proliferation, migration and collagen metabolism were associated with the Dkk1/Wnt and NF-κB signaling pathways in hypoxia-induced PVR.

In 2020, Zhang (Zhang et al., 2021) first identified the endogenous SO_2_/AAT1 pathway in MCs. To further investigate whether cAMP mediates the inhibitory effect of endogenous SO_2_ on MC degranulation, researchers used the PDE inhibitor IBMX or the AC activator forskolin to increase cAMP level in MCs and discovered that IBMX and forskolin successfully terminated MC degranulation induced by AAT1 knockdown. Additionally, in hypoxia-stimulated MCs, AAT1 protein expression and SO_2_ production were significantly downregulated, resulting in the activation of MC degranulation. However, AAT1 overexpression, which increased endogenous SO_2_ levels, inhibited MC degranulation, and this inhibitory effect was disrupted by the cAMP synthesis inhibitor SQ22536. The results from animal experiments supported these findings and showed that the mRNA and protein expression of AAT1 significantly decreased in rat lung tissue under hypoxia. Treatment with SO_2_ donor increased cAMP level in the lung tissue of hypoxic rats and reduced the accumulation and degranulation of MCs around the pulmonary arteries. These studies suggested that SO_2_ acted as an endogenous stabilizer of MCs by upregulating the cAMP pathway to inhibit MC degranulation. Given the significant role of MC degranulation in pulmonary vascular inflammation and PVR, inhibition of MC degranulation may be an essential mechanism by which MC-derived SO_2_ suppresses hypoxia-induced PVR.

### 4.2 High pulmonary blood flow-induced CHD-PH model

The endogenous SO_2_ system is involved in the development of PH induced by high pulmonary blood flow. Luo et al. investigated the role of the SO_2_/AAT2 pathway in the pathogenesis of PH induced by high pulmonary blood flow in 2013. The study revealed that administering SO_2_ donor alleviated the elevation in pulmonary arterial pressure caused by high pulmonary blood flow and reduced the degree of pulmonary arterial muscularization. Additionally, SO_2_ donor increased the production of H_2_S in rat lung tissue, upregulated the protein and mRNA expressions of CSE, and increased 3-MPST and cystathionine β-synthase (CBS) mRNA levels (Luo et al., 2013). These results suggested that SO_2_ reduced PH induced by high pulmonary blood flow and exerted a protective effect against pulmonary vascular pathology, which was associated with the upregulation of the endogenous H_2_S pathway.

Liu et al. (2016) investigated the role of the endogenous SO_2_/AAT1 pathway in collagen synthesis in the fibroblasts of rats with high pulmonary blood flow-induced PH. These results demonstrated that reduced endogenous SO_2_ level promoted collagen accumulation in the lung tissues of rats with shunt-induced PH. Further mechanistic studies revealed that endogenous SO_2_ inhibited the activation of the TGF-β1 signaling pathway in PAFs, consequently suppressing pulmonary vascular collagen synthesis. In a model of mechanical stretching-induced PAF collagen synthesis, AAT1 overexpression increased endogenous SO_2_ production and inhibited TGF-β1 expression and Smad2/3 phosphorylation. Conversely, AAT1 knockdown simulated the effects of mechanical stretching, leading to increased TGF-β1 expression and Smad2/3 phosphorylation. Additionally, the use of the TGF-β1/Smad2/3 signaling pathway inhibitor SB431542 eliminated the excessive accumulation of collagen I and collagen III induced by AAT1 knockdown, mechanical stretch or a combination of both. Furthermore, in a rat model of pulmonary vascular collagen accumulation induced by high pulmonary blood flow, supplementation with SO_2_ donor inhibited the activation of TGF-β1/Smad2/3 signaling pathway and alleviated excessive collagen deposition in the lung tissue of rats with shunt-induced PH. These findings indicated that the endogenous SO_2_/AAT1 pathway could inhibit the activation of the TGF-β1/Smad2/3 signaling pathway to suppress the abnormal collagen accumulation induced by mechanical stretching in PAFs.

### 4.3 MCT-induced PH model

Endogenous SO_2_ exerts an inhibitory effect on MCT-induced redox injury and collagen synthesis in the lung tissue, thereby antagonizing MCT-induced PH formation. In 2008, Jin et al. (2008) investigated the regulatory role of endogenous SO_2_ in a rat model of MCT-induced PH and administered HDX to inhibit the upregulation of the SO_2_/AAT1/2 pathway induced by MCT. The results showed that compared to the MCT group, rats treated with MCT + HDX exhibited significantly increased mPAP and PVR and a significant decrease in lung tissue antioxidant substances. However, intervention with SO_2_ donor significantly reduced PVR in rats and increased the levels of antioxidant substances in the lung tissue. These findings suggest that endogenous SO_2_ enhanced the antioxidant capacity of lung tissue and inhibited MCT-induced PVR and PH. In 2016, Yu et al. (Yu et al., 2016) investigated the role of endogenous SO_2_ in MCT-induced pulmonary vascular collagen synthesis and revealed that the inhibition of endogenous SO_2_ generation in the lung tissue by HDX further aggravated MCT-induced collagen synthesis in rat pulmonary vessels. Conversely, supplementation with SO_2_ donor suppressed MCT-stimulated collagen synthesis and increased collagen degradation to alleviate pulmonary vascular collagen remodeling.

Mechanistic insights revealed that SO_2_ donor downregulated the expression of TGF-β1 induced by MCT in pulmonary arteries and alleviated pulmonary vascular collagen remodeling, suggesting the involvement of TGF-β1-related signaling pathways in the protective effect of SO_2_. Further investigations using cultured PAFs stimulated with TGF-β1 have shed light on the mechanisms underlying the effects of AAT1 overexpression in promoting an increased endogenous SO_2_ level and preventing the activation of the TGF-β/TβRI/Smad2/3 signaling pathway, as well as abnormal collagen synthesis in PAFs. Conversely, in TGF-β1-treated PAFs, AAT1 knockdown reduced endogenous SO_2_ level, exacerbated Smad2/3 phosphorylation, and collagen I and collagen III deposition (Yu et al., 2016). These results supported the notion that endogenous SO_2_ inhibited the activation of the TGF-β/TβRI/Smad2/3 signaling pathway in PAFs, thereby suppressing MCT-induced collagen synthesis in rat pulmonary arteries.

## 5 Significance and prospects of SO_2_ in PH

Taken together, insufficient endogenous production of SO_2_ has been recognized as an important pathogenic mechanism underlying PH and PVR. The SO_2_/AAT pathway serves as a crucial endogenous regulatory pathway in PAECs, PASMCs, PAFs, macrophages, MCs and other cell types. Moreover, molecular SO_2_, HSO_3_^−^, SO_3_^2−^ and proteins containing -S (=O)-OH in body fluids may act as a whole controlled by a complex and incompletely elucidated metabolic regulatory network (Chen et al., 2017; Song et al., 2020; Stipanuk, 2020; Huang et al., 2022). The degree of downregulation of HSO_3_^−^ and SO_3_^2−^ can partly reflect the severity of CHD-PH (Yang et al., 2014). Supplementation with SO_2_ donor in animal models of PH induced by hypoxia, monocrotaline or high pulmonary blood flow significantly inhibited PVR by attenuating inflammation, proliferation and collagen dysregulation in the pulmonary vascular wall; thus, preventing the development of PH (Jin et al., 2008; Sun et al., 2010; Luo et al., 2013).

Previous clinical treatment strategies for PH have primarily focused on the excessive vasoconstriction of pulmonary blood vessels. However, the structural damage to blood vessels limits the efficacy of vasodilator drugs (Shah et al., 2023). Nevertheless, the successful application of NO, the first gaseous signaling molecule used in the treatment of PH, provides a positive reference for the treatment of clinical PH with SO_2_ (Yu et al., 2019). To date, there have been few assessments of the therapeutic effects of SO_2_ on clinical PH and the limitations of its clinical application. Given the strong and pungent odor of SO_2_ gas, along with its unsafe storage, high price, and inconvenient portability, the most commonly used SO_2_ donor is a mixture of HSO_3_^−^ and SO_3_^2−^. However, the emergence of SO_2_ prodrugs that can simulate the slow release of endogenous SO_2_ *in vivo* and can bound lipid nanoparticles targeting the lungs, would make it possible to enhance the pulmonary specificity and stability of SO_2_ protection against vascular remodeling through inhaled prodrugs, and to avoid the side effects of systemic hypotension (Wang et al., 2018; Li et al., 2020; Yu et al., 2020; Huang et al., 2021; Huang et al., 2022; Li et al., 2023). In addition, the development of small molecule activators for SO_2_ synthase also has broad prospects. In general, considering that molecular SO_2_, HSO_3_^−^, SO_3_^2−^ and proteins containing -S (=O)-OH may act together as a whole, further exploration of the regulatory role and mechanisms of SO_2_ in PH and PVR, and the development of SO_2_ prodrugs holds the potential to utilize SO_2_-related medications to inhibit or reverse PVR and would improve the clinical treatment efficacy and quality of life for patients with PH-related diseases.
