# Supplementary material for: Advances in the research of sulfur dioxide and pulmonary hypertension
Source: Front Pharmacol. 2023 Oct 12;14:1282403. doi: 10.3389/fphar.2023.1282403 (PMC10602757; doi:10.3389/fphar.2023.1282403)
Supplement: Supplementary file 1 [file Table1.docx]

Advances in the Research of Sulfur Dioxide and Pulmonary Hypertension

Xin Liu ^1^, He Zhou ^2^, Hongsheng Zhang ^1^, Hongfang Jin ^3,^* and Yan He ^1,^*

^1^ Department of Pediatric Cardiac Center, Beijing Anzhen Hospital, Capital Medical University, Beijing 100029, China; LX: 18811563661@163.com; ZHS: zhanghongshengyou@126.com; HY: hybicq@qq.com

^2^ Departments of Medicine and Physiology, Tulane University School of Medicine, New Orleans, LA, 70112, USA; hzhou5@tulane.edu

^3^ Department of Pediatrics, Peking University First Hospital, Beijing 100034, China; jinhongfang51@126.com

***** Correspondence: JHF: jinhongfang51@126.com; HY: hybicq@qq.com

**Supplementary Table 1.**

**Effects of sulfur dioxide on pathophysiological processes of the pulmonary vasculature and aorta.**

| **Pathophysiological processes** | **Pulmonary** **vasculature** | **Aorta** |
| --- | --- | --- |
| Vasomotor function | Not assessed | Vasorelaxation |
| Inflammation | Inhibition | Not assessed |
| Oxidative stress | Inhibition | Inhibition |
| Vascular remodeling | Inhibition | Inhibition |
| EC-apoptosis | Inhibition | Not assessed |
| SMC-apoptosis | Not assessed | Promotion |
| SMC-proliferation | Inhibition | Inhibition |
| Collagen metabolism | Inhibition | Inhibition |

EC: endothelial cell, SMC: smooth muscle cells.

References

1. Lu W, Sun Y, Tang C, Ochs T, Qi J, Du J, Jin H. Sulfur dioxide derivatives improve the vasorelaxation in the spontaneously hypertensive rat by enhancing the vasorelaxant response to nitric oxide. Experimental biology and medicine (Maywood, NJ). **2012**;237(7):867-72.

2. Liu X, Zhang S, Wang X, Wang Y, Song J, Sun C, Chen G, Yang G, Tao Y, Hu Y, Bu D, Huang Y, Du J, Jin H. Endothelial Cell-Derived SO_2_ Controls Endothelial Cell Inflammation, Smooth Muscle Cell Proliferation, and Collagen Synthesis to Inhibit Hypoxic Pulmonary Vascular Remodelling. Oxidative medicine and cellular longevity. **2021**;2021:5577634.

3. Shen Z, Huang P, Du S, Li K, Yu X, Tang C, Du J, Jin H. Effects of endogenous sulfur dioxide on the oxidative stress induced by cobalt chloride in the rat pulmonary artery smooth muscle cells. Chin J Appl Clin Pediatr. **2017**;32:672–676.

4. Li W, Tang C, Jin H, Du J. Regulatory effects of sulfur dioxide on the development of atherosclerotic lesions and vascular hydrogen sulfide in atherosclerotic rats. Atherosclerosis. **2011**;215(2):323-30.

5. Liu X, Zhang D, Li K, Yu X, Tang C, Du J, Jin H, Huang Y. Effect of endogenous sulfur dioxide on the apoptosis induced by cobalt chloride in the human pulmonary arterial endothelial cells. Chin J Appl Clin Pediatr. **2018**;33: 999–1003.

6. Zhao X, Jin HF, Tang CS, Du JB. [Effects of sulfur dioxide, on the proliferation and apoptosis of aorta smooth muscle cells in hypertension: experiments with rats]. Zhonghua yi xue za zhi. **2008**;88(18):1279-83.

7. Wu HJ, Huang YQ, Chen QH, Tian XY, Liu J, Tang CS, Jin HF, Du JB. Sulfur Dioxide Inhibits Extracellular Signal-regulated Kinase Signaling to Attenuate Vascular Smooth Muscle Cell Proliferation in Angiotensin II-induced Hypertensive Mice. Chinese medical journal. **2016**;129(18):2226-32.

8. Yu W, Liu D, Liang C, Ochs T, Chen S, Chen S, Du S, Tang C, Huang Y, Du J, Jin H. Sulfur Dioxide Protects Against Collagen Accumulation in Pulmonary Artery in Association with Downregulation of the Transforming Growth Factor β1/Smad Pathway in Pulmonary Hypertensive Rats. Journal of the American Heart Association. **2016**;5(10).

9. Liu J, Yu W, Liu Y, Chen S, Huang Y, Li X, Liu C, Zhang Y, Li Z, Du J, Tang C, Du J, Jin H. Mechanical stretching stimulates collagen synthesis via down-regulating SO_2_/AAT1 pathway. Scientific reports. **2016**;6:21112.

10. Huang Y, Li Z, Zhang L, Tang H, Zhang H, Wang C, Chen SY, Bu D, Zhang Z, Zhu Z, Yuan P, Li K, Yu X, Kong W, Tang C, Jung Y, Ferreira RB, Carroll KS, Du J, Yang J, Jin H. Endogenous SO_2_-dependent Smad3 redox modification controls vascular remodeling. Redox biology. **2021**;41:101898.
